# Equity Gaps in Epistemic Cognition Between Black, Hispanic/Latino, Asian, and White University Students

**DOI:** 10.3390/bs16071217

**Published:** 2026-07-17

**Authors:** Julia Zavala, Rebecca C. Trenz, Terrence Calistro, Deanna Kuhn

**Affiliations:** 1 Department of Psychology, Mercy University, Dobbs Ferry, NY 10522, USA; tcalistro@mercy.edu; 2 Department of Psychology, Clarkson University, Potsdam, NY 13699, USA; rtrenz@clarkson.edu; 3 Department of Human Development, Teachers College, Columbia University, New York, NY 10027, USA; dk100@tc.columbia.edu

**Keywords:** epistemic cognition, equity gaps, underrepresented minority students, university students

## Abstract

Previous research has shown that epistemic cognition—the knowledge about what makes a belief justified or true—tends to be higher among White students compared to Black, Hispanic/Latino, and Asian students. Much of this research has been done using the independent dimensions model of epistemic cognition and only one study included a relatively small proportion of Hispanic/Latino participants in STEM. Using the developmental model of epistemic cognition that consists of three levels—absolutist, multiplist, and evaluativist—a sample of university students from a single institution with diverse majors and racial and ethnic backgrounds completed the Epistemic Thinking Assessment. Subsequently, they were categorized into one of the three levels of epistemic cognition. The results showed that Black and Hispanic students were more likely to have absolutist as their predominant epistemic cognition level and less likely to have evaluativist as their predominant epistemic cognition level relative to White students despite family income. The effect sizes in the regression models are small. Future research should explore how social and environmental factors may contribute to these results. Furthermore, the findings illuminate an opportunity for interventions that can be tested to determine if they support the development of epistemic cognition and close equity gaps, such as evidence-based pedagogies rooted in developing argumentation skills.

## 1. Introduction

Understanding equity gaps among underrepresented minority groups is an important endeavor in higher education in the United States as closing achievement gaps has been essential to the guiding principles in the analysis of the accreditation standards set by the Middle States Commission on Higher Education (2023). Educators and institutions in higher education have been focused on closing equity gaps in graduation, retention rates, and academic achievement. The resources and skills that students come to college with vary, and can impact equity gaps in the aforementioned outcomes. One in particular is epistemic cognition, which is the “cognition of or relating to knowledge” (Greene et al., 2016, p. 2), more specifically defined as “knowledge about matters of epistemology—that is, knowledge about the justification and truth of beliefs” (Moshman, 2015, p. 144). The literature has yet to fully examine equity gaps in epistemic cognition. The value in understanding equity gaps in epistemic cognition lies in its relationship to academic achievement (Greene et al., 2018) which in turn is essential to retention (Sass et al., 1970/2018). Furthermore, epistemic cognition is a key component in critical thinking (Hofer, 2016; Kuhn, 2005; Kuhn et al., 2000; Kuhn & Weinstock, 2002), reasoning, and rationality (Kuhn, 2005; Moshman, 2015) and these constructs are necessary for success in the workplace (U.S. Department of Labor, n.d.) and meaningful global citizenship (Kaya, 2022). Essentially, epistemic cognition is foundational to sustaining academic and non-academic success.

### 1.1. Theoretical Framework for the Developmental Sequence of Epistemic Cognition

The study of epistemic cognition as a developmental sequence originates from Perry’s (1970/1999) seminal research at Harvard University, conducted with primarily White male students (Hofer, 2016; Hofer & Pintrich, 1997; Moshman, 2015; Perry, 1970/1999). From student interviews about their experiences in college, Perry identified nine positions grouped into four hierarchical categories: dualism, multiplicity, contextual relativism, and commitment within relativism. Dualism reflects belief in absolute right and wrong and reliance on authority for truth. Multiplicity involves recognition of uncertainty, with later stages emphasizing a right to individual opinion. Relativism introduces contextual understanding, where knowledge is seen as constructed and evaluated based on evidence. Commitment within relativism focuses less on epistemic cognition and rather extends to personal identity, responsibility, and values. Although Perry did not label this sequence as epistemic development, his work provided foundational evidence for a developmental model of epistemic cognition in adulthood.

Subsequent theorists have refined Perry’s ideas, generally agreeing that epistemic cognition progresses from objectivism (dualism) to subjectivism (multiplicity) to rationalism (relativism), with the latter reflecting increasing coordination between objective and subjective knowing (Moshman, 2015). Perry’s foundational work led to Kuhn’s model of “epistemological understanding” (Kuhn, 1991, 2005; Kuhn et al., 2000; Kuhn & Weinstock, 2002), which is applied in the present study and similarly outlines three levels: absolutist, multiplist, and evaluativist. At the absolutist level, knowledge is viewed as objective, certain and externally derived, in which critical thinking consists of simply comparing assertions to reality to determine if they are true or not. At the multiplist level, knowledge is viewed as subjective opinion that dismisses evaluation, where critical thinking is irrelevant as everyone is considered to have a right to their own opinion. Lastly, at the evaluativist level, one integrates subjectivity with evidence-based objective reasoning, recognizing that some claims are better justified than others. At this stage, knowledge is viewed as constructed and open to revision, with critical thinking used to enhance understanding (Kuhn, 2005).

Together, Perry’s and Kuhn’s frameworks establish epistemic cognition as a developmental process in which individuals progress from viewing knowledge as fixed to understanding it as contextual, constructed, and justifiable.

### 1.2. The Developmental Model Compared to the Multiple Independent Dimensions Model

Although variation in epistemic cognition across and within cultures has received limited attention, existing studies have largely measured epistemic cognition as a set of independent dimensions (Karabenick & Moosa, 2005; Schommer-Aikins & Easter, 2008; Strømsø et al., 2016) rather than as a developmental process (Kuhn & Park, 2005; Thompson et al., 2023). For example, Schommer-Aikins (1990) introduced a multidimensional model of epistemic cognition consisting of five factors: fixed ability, quick learning, simple knowledge, certain knowledge, and source of knowledge. According to this perspective, epistemic beliefs can vary independently rather than following a unidimensional developmental progression. However, due to the fact that multiple dimensions can co-occur at different levels, this approach cannot track developmental change over time.

Subsequent research has refined these conceptual distinctions. Hofer and Pintrich (1997) argued that fixed ability and quick learning are not constructs of epistemic cognition and the remaining dimensions—simplicity, certainty, and source of knowledge—align with developmental models as individuals progress through the absolutist, multiplist, and evaluativist levels. Further supporting this developmental view, Barzilai and Weinstock (2015) created a semantic differential scale capturing dimensions of certainty and source/justification within each of these three levels.

Building on these frameworks, the present study employs Barzilai and Weinstock’s (2015) measure to assess participants’ predominant epistemic cognition level and investigates racial and ethnic equity gaps within a developmental, rather than multidimensional, model of epistemic cognition.

### 1.3. Cultural and Racial Differences in Epistemic Cognition

Research on racial and cultural differences in epistemic cognition has yielded mixed results. Karabenick and Moosa (2005) compared epistemological beliefs about science between Omani and U.S. students (the latter predominantly White) and found that Omani students viewed knowledge as simpler and more certain, whereas U.S. students regarded it as complex and evolving. Within the U.S., Schommer-Aikins and Easter (2008) observed that European Americans were more inclined to believe that it takes time to acquire knowledge and that knowledge may be complex and structured in an integrated way in comparison to Asian Americans, and demonstrated stronger links between epistemic beliefs and academic performance.

Similarly, Strømsø et al. (2016) investigated epistemological beliefs among ethnic minority and majority Norwegian high school students. Although no significant group differences were found overall, ethnic minority students who relied on authority-based justification showed higher topic knowledge and comprehension, suggesting that deference to authority may function as a learning strategy in some cultural contexts.

Few studies, however, have examined epistemic development across cultural or racial groups. Kuhn and Park (2005) compared Caucasian, Korean, and Japanese parent–child dyads and found higher frequency of evaluativist epistemic cognition among Caucasian-Americans, indicating potential cultural variation in epistemic development. Expanding on this developmental perspective, Thompson et al. (2023) analyzed epistemic trajectories among 4500 undergraduate STEM students from 14 U.S. universities. Hispanic and non-native English-speaking students were more likely to remain absolutist, Black students more likely to remain multiplist, and those who advanced to the evaluativist stage were more often native English speakers and of “Other race.”

Collectively, these studies demonstrate that epistemic cognition may be shaped by cultural and racial contexts. Furthermore, most prior work has treated epistemic cognition as independent constructs rather than part of a developmental progression, a gap the present study seeks to address.

### 1.4. Present Study

Given the mixed findings and the methodologically heterogenous measures of epistemic cognition in the reviewed literature, the present study aims to further understand and explore equity gaps in epistemic cognition by using the developmental model to compare epistemic cognition level of students identifying as Asian, Black/African American, or Hispanic/Latino relative to students identifying as White from a university in the U.S. In the current study, White students served as the reference group as the above literature identified differences in epistemic cognition between White, Asian, and Black participants and other racially diverse groups. Additionally, those who identified as White in the current study made up the second largest group in the sample (*n* = 111, 22.7%). 

Additionally, Thompson et al. (2023) focused on undergraduate STEM students from R1 universities and the proportion of Black (6%) and Hispanic students (8%) in their study is small relative to White students (61%). The current study explores these relationships beyond STEM and includes undergraduate and graduate students from a Hispanic and Minority Serving Institution, providing a holistic picture of these relationships among a less research-focused population of college students. This study will include a larger proportion of Hispanic and Black students. Overall, understanding the racial and ethnic gaps in epistemic cognition is critical so that educators can intervene and work to close equity gaps using evidence-based educational pedagogies.

The research questions that are addressed in this study are as follows:

1. What demographic variables are associated with the three levels of epistemic cognition (absolutist, multiplist, and evaluativist)?
2. Is race/ethnicity associated with the absolutist level while controlling for demographic variables?
3. Is race/ethnicity associated with the multiplist level while controlling for demographic variables?
4. Is race/ethnicity associated with the evaluativist level while controlling for demographic variables?

## 2. Materials and Methods

### 2.1. Participants and Procedure

A total of 490 college students were included in this study (Mage = 22.66, SDage = 6.79). The majority were female (70%). Most participants identified as Hispanic/Latino (45.50%), White (22.70%), and Black (19.60%). See Table 1 for full demographic data. This study is a secondary data analysis of data collected from June 2019 through July 2020 using convenience sampling. Prior to March 2020, participants that were recruited for the original study were recruited in-person, via classroom visits, email, social media, SONA (a research participation software; https://www.sona-systems.com/, MD, USA), and flyers posted throughout campus. After March 2020, participants were only recruited via email, social media, and SONA. Participants provided consent then completed the survey. Those who participated in person received $5. Those who participated online were entered into a raffle to receive one of 50 $20 Amazon gift cards. Students who were taking it for extra credit or research participation credit were not monetarily remunerated. On average, participants took 20 min to complete the questionnaires. Forty-one participants completed the survey in person and the remaining completed the study online via Redcap (https://project-redcap.org/, TN, USA). For the purposes of this study, only data from the Epistemic Thinking Assessment and the demographic questionnaire were examined.

### 2.2. Materials

#### 2.2.1. Epistemic Cognition

To measure epistemic cognition, the Epistemic Thinking Assessment (ETA) was used (Barzilai & Weinstock, 2015). It is a quantitative measure of epistemic perspectives. It consisted of a fictitious history scenario, titled The Fifth Livian War, which included a brief background of the war and two accounts from expert historians of the war (adapted from Kuhn & Weinstock, 2002). The assessment was made up of 12 questions. Each question included 3 responses that asked the individual to indicate how much they agree with the given statement on a 10-point scale from 1 ‘very much disagree’ to 10 ‘very much agree’. Each response was indicative of either an absolutist, multiplist, or evaluativist perspective. Mean scores for the 3 levels were calculated to assess agreement with each epistemic perspective. Then the highest score was used to categorize participants into one of the three epistemic levels—absolutist, multiplist, or evaluativist. Barzilai and Weinstock found the ETA to be a reliable and valid measure of epistemic thinking with construct and internal consistency reliabilities for each epistemic perspective above 0.70. Internal consistency reliability based on Cronbach’s alphas was also found within this study for each epistemic level: α = 0.81 for absolutist, α = 0.83 for multiplist, and α = 0.81 for evaluativist. Validity was established by Barzilai and Weinstock (2015), where they found that the scores for the 3 epistemic perspectives from the history scenario, which was used in the present study, were highly correlated with scores from a scientific scenario. 

#### 2.2.2. Demographic Questionnaire

Participants responded to a demographic questionnaire that included age, year in college, major, grade point average (GPA), employment status (yes or no), gender (female, male, or do not wish to specify), ethnicity (Asian, Black or African American, Hispanic or Latino, Native American, Native Hawaiian or Pacific Islander, White, other, biracial, or multiracial), and family income. Several majors existed, so they were categorized based on similar fields.

### 2.3. Data Analysis Plan

Chi-square tests for independence were conducted to determine if relationships existed between the categorical demographic variables (major, year in college, family income [less than $75,000 or $75,000 or more; responses where participants selected “Don’t know/Not sure” or “Decline to respond” were excluded from analysis, *n* = 174 (35.5%)], and race/ethnicity [Asian including Native Hawaiian or Pacific Islander, Black or African American, Hispanic or Latino, and White]); and the three levels of epistemic cognition (absolutist, multiplist, and evaluativist). Levels of epistemic cognition were dichotomized and correlated with age and GPA using point-biserial correlations which examine associations between a numeric/continuous variable and a binary/dummy coded variable (Kornbrot, 2014). Lastly, three hierarchical binary logistic regressions were conducted to examine how race/ethnicity was associated with the three levels of epistemic cognition while controlling for the demographic variable(s) related to epistemic cognition from the aforementioned analyses. Binary logistic regressions were preferred over multinomial logistic regressions due to the small number of participants who identified as multiplist (*n* = 29).

## 3. Results

Participants were categorized into one of the three epistemic cognition levels—absolutist, multiplist, or evaluativist—based on the predominant mean score across the three levels. Table 2 provides the overall mean scores for each level. Categorizing epistemic cognition into a predominant level is a common practice among scholars examining the development of epistemic cognition (Kuhn & Weinstock, 2002; Thompson et al., 2023; Zavala & Kuhn, 2017). The present study showed that 50% of the participants were categorized at the absolutist level, 37.10% at the evaluativist level, and 5.90% at the multiplist level. See Table 2. Thirty-four (6.9%) of the participants had similar scores for some of the levels.

Four chi-square tests of independence were calculated to examine the relationships between epistemic cognition levels and major, academic year, household income, and race/ethnicity. Levels of epistemic cognition were not associated with major ($\chi^{2}$ (12) = 8.35, *p* = 0.757, *V* = 0.14); therefore, all majors were combined in subsequent regression analyses. Additionally, epistemic cognition levels were not associated with academic year ($\chi^{2}$ (10) = 11.96, *p* = 0.288, *V* = 0.16); therefore, undergraduate and graduate students were grouped together in subsequent analyses. Levels of epistemic cognition were shown to have significant associations with household income ($\chi^{2}$ (2) = 13.03, *p* = 0.001, *V* = 0.21), and race ($\chi^{2}$ (6) = 23.28, *p* < 0.001, *V* = 0.24). Participants categorized as evaluativist had lower odds of having a family income less than $75,000 (32.7%) than those categorized as absolutist (57.2%). Participants who were Hispanic (57.1%) and Black (58.6%) were associated with greater odds of predominantly being at the absolutist level of epistemic cognition compared to participants who were White (40.7%) and Asian (40%).

The Pearson correlation coefficients that were calculated to assess the associations between age, cumulative GPA, and epistemic cognition (absolutist, multiplist, and evaluativist) were not significant at the 0.05 level of significance. Therefore, in the subsequent analyses, age and GPA did not serve as control variables.

Three hierarchical binary logistic regression models were conducted to examine the likelihood of race/ethnicity being associated with each epistemic cognition level while controlling family income. Students identifying as White were set as the reference group as the literature identified differences in epistemic cognition between White, Asian, and Black participants and other racially diverse groups. Also, this group was the second largest group in the sample. In terms of the absolutist level, despite the relatively low $R^{2}$ in this model ($R^{2}$ = 0.06) indicating that the race categories explain a small amount of variance in the dependent variable, the results showed that Black (OR = 2.37, *p* = 0.020) and Hispanic participants (OR = 2.57, *p* = 0.002) were associated with greater odds of absolutist being their predominant level of epistemic cognition relative to participants who identified as White. See Table 3. Race/ethnicity was not shown to statistically relate to the multiplist level. In this model the $R^{2}$ value is low ($R^{2}$ = 0.05) and confidence intervals were wide for race categories. See Table 4. In terms of the evaluativist level, the $R^{2}$ value ($R^{2}$ = 0.11) and confidence intervals are adequate, and the results showed that Black (OR = 0.34, *p* = 0.005) and Hispanic participants (OR = 0.31, *p* < 0.001) were associated with lower odds of evaluativist being their predominant level of epistemic cognition relative to participants who identified as White. See Table 5. Given the small $R^{2}$ values and wide confidence intervals for some variables, these results should be interpreted with caution. 

## 4. Discussion

The current study aimed to understand how race/ethnicity was associated with the three levels of epistemic cognition (absolutist, multiplist, and evaluativist) while controlling for demographic variables among university students from a single institution. Half of the participants were predominantly absolutist and just over a third were at the evaluativist level. Study findings showed that four demographic variables, age, cumulative GPA, major, and year in college were not associated with levels of epistemic cognition, but family income was. Furthermore, students who were Hispanic or Latino were more likely to be at the absolutist level of epistemic cognition and less likely at the evaluativist level compared to students who were White despite family income. These results align with Thompson et al. who also found that students at the absolutist level were more likely to be Hispanic (Thompson et al., 2023).

Moreover, the current research found that Black and African American students were also more likely to be absolutist and less likely to be evaluativist relevant to their counterparts who identified as White. This finding contrasts with Thompson et al. (2023) who found that Black undergraduate STEM students were more likely to be at the multiplist level. The current study includes students from majors in STEM and beyond, which helps to avoid limiting the results to students in only one specific type of major. Therefore, these findings suggest that the epistemic cognition level for Black students across several majors in this study may be absolutist. Furthermore, race/ethnicity was not associated with the multiplist level while controlling for family income. This may be due to the low number of participants that were categorized as multiplist and the unstable estimates in the regression. These results, particularly for the multiplist group, should be interpreted with caution. 

Systemic structural environments that individuals live in as well as the systemic implicit biases that they experience were not directly measured in this study, but future research could examine these further to understand how these constructs may impact equity gaps in epistemic cognition. For instance, a greater percentage of Black (17%) and Hispanic (13%) individuals living in the United States live in high-poverty neighborhoods compared to Asian and White individuals (4% each) (National Equity Atlas, 2022). Furthermore, educational funding at the local level is lowest in impoverished neighborhoods but this has been counteracted by the redistribution of funds at the state and federal level, resulting in no significant difference in funding between poor and non-poor districts (Chingos & Blagg, 2017). However, this continues to be troubling due to the lack of access to resources students in poor districts may have to supplement additional resources and this can negatively impact academic success (National Association of Secondary School Principals, n.d.).

In addition to a lack of resources, the role of implicit biases on equity gaps in epistemic cognition could be explored by future research. Historically, underrepresented minorities have been treated differently in educational settings and data show that this has not changed. Recently, researchers have found that Black children are more harshly punished compared to White students and non-Black students (Darling-Hammond & Ho, 2024). They are more likely to have “in-school suspensions, out-of-school suspensions, expulsions, corporal punishment, referrals to law enforcement, or school-related arrests” (Darling-Hammond & Ho, 2024, p. 11) regardless of gender and the type of school attended. Furthermore, Latinx students are also disciplined more harshly than their White counterparts (Gage et al., 2021). This disproportionate punishment may lead to a demoralizing outlook of the educational system and could be demotivating for individuals (Darling-Hammond, 2023).

Once they have entered college, earlier educational experiences may have shaped students’ epistemic cognition. However, epistemic cognition is malleable and develops with sustained practice and interventions. For instance, college students who engaged in a one-time intervention where they constructed an argumentive dialogue demonstrated higher levels of epistemic cognition compared to their counterparts in a control group (Zavala & Kuhn, 2017). Furthermore, changes in epistemic cognition can be more effective on those with more rudimentary epistemic cognition (Kerwer & Rosman, 2020). Future studies should aim to replicate these findings and test whether equity gaps could be closed by interventions that support the development of metacognitive and argument skills. Specifically, these interventions should focus on exposing students to both sides of a problem, encouraging the use of evidence to support a claim, and supporting the development of counterarguments to defend a position.

This research is exploratory and is limited by the fact that it is a secondary data analysis that used a cross-sectional design. This led to small numbers in racial/ethnic groups and these results need to be interpreted with caution. However, this design was necessary in order to understand whether equity gaps existed in the first place among Black, Latino, Asian, and White college students from a diverse institution in the U.S. This study used convenience sampling from a single institution which limits the generalizability of the findings to college students beyond the current sample. Despite this limitation, it contributes to the literature by including approximately two-thirds of non-White students in the analysis, which reflects the demographic make-up of the Hispanic/minority serving institution. Additionally, 34 participants were not classified into one predominant epistemic cognition level as they had similar scores for two or all three of the levels. Future research should collect qualitative responses to use as a secondary source to help classify participants into one level or to better understand if there is a potential transition being made from one epistemic cognition level to the next. Moreover, classifying participants into one level is an analytical simplification and may limit the ability to identify important within-subject variation across absolutist, multiplist, and evaluativist epistemic cognition levels. Data was collected in 2019–2020 and may reflect epistemic cognition prior to and during the COVID-19 pandemic. Many students participated in online learning which may have impacted their epistemic cognition. However, it could serve as a marker of equity gaps in epistemic cognition during that time for researchers aiming to replicate this post-pandemic and track changes that may have occurred. Another limitation to this study is collapsing heterogenous identities into one broad category such as Asian or Hispanic/Latino. These identities are made up of different cultural identities that may vary in many ways. Future research should account for this by asking participants to select from a more nuanced list of identities. Participants who identified as Asian (*n* = 19) were aggregated with participants who identified as Native Hawaiian/Pacific Islander (*n* = 4) to create a larger group for analysis. This replicated the group makeup demonstrated in Thompson et al. (2023), but may mask meaningful within-group disparities. This study was a secondary data analysis and we were limited by the number of participants in each group; future work should aim to collect a larger number of participants in each category so they may be disaggregated and better represented. Furthermore, the literature suggests that race/ethnicity intersects with factors such as socioeconomic status, language background, and first-generation status (Liggett, 2009; Lundberg et al., 2007; National Equity Atlas, 2022). Given that this study was a secondary data analysis, we did not have data for these potential intersecting variables available to us except for family income which was used as a control variable. This limits the ability to understand how other variables may interact with race/ethnicity to impact epistemic cognition. We controlled for socioeconomic status; however, future research should explore how intersecting variables moderate the relationship between race/ethnicity and epistemic cognition levels. Lastly, just over a third of the participants had to be excluded for not knowing or not choosing to report their family income. This may obscure the true impact of family income on epistemic cognition when race/ethnicity are considered.

## 5. Conclusions

This study adds to the existing literature as the findings show that equity gaps exist in epistemic cognition among a sample of Black, Latino, Asian, and White college students from a single institution in the U.S., an area of research that has not been extensively studied. Only one other research study has examined equity gaps across these demographics where the focus was primarily on undergraduate STEM students (Thompson et al., 2023). The current study examines undergraduate and graduate students in a broader array of majors and includes a higher proportion of underrepresented minority students. Future research should further explore the causes of these gaps as they may be rooted in the neighborhoods individuals grow up in and the biases they may experience. Additionally, future research should test whether interventions focusing on argumentation and metacognitive skills close these gaps in epistemic cognition. 

## Figures and Tables

**Table 1:** Demographic table.

Variable		*M*	*SD*
Age		22.66	6.79
GPA		3.25	0.48
Variable		*n*	%
Do You Work?	No	167	34.10%
	Yes	323	65.90%
Gender	Male	139	28.40%
	Female	343	70%
Race/Ethnicity	Asian (includes East Asian, South Asian, and Middle Eastern)	19	3.90%
	Black or African American	96	19.60%
	Hispanic or Latino	223	45.50%
	Native American	1	0.20%
	Native Hawaiian or other Pacific Islander	4	0.80%
	White	111	22.70%
	Bi-racial	20	4.10%
	Multi-racial	6	1.20%
	Other	10	2%
Family Income	<$25,000	82	16.70%
	$25,000–<$50,000	85	17.30%
	$50,000–<$75,000	61	12.40%
	$75,000–<$100,000	32	6.50%
	$100,000–<$150,000	31	6.30%
	≥$150,000	25	5.10%
	Don’t know/Not sure	116	23.70%
	Decline to respond	58	11.80%
Year in College	Freshman	188	38.4%
	Sophomore	71	14.5%
	Junior	85	17.3%
	Senior	99	20.2%
	Masters	46	9.4%
	Doctorate	1	0.2%
Major	Arts, Humanities, & Communication	31	6.4%
	Business & Management	44	9.1%
	Computers & Technology	20	4.1%
	Criminal Justice & Law	53	10.9%
	Health & Natural Sciences	156	32.1%
	Social Sciences & Education	154	31.7%
	Other/Interdisciplinary	28	5.8%

**Table 2:** Distribution of participants across levels of epistemic cognition.

Variable		*n*	%	*M* (*SD*)
Epistemic Cognition Level	Absolutist	245	50%	6.63 (1.35)
	Multiplist	29	5.90%	4.62 (1.46)
	Evaluativist	182	37.10%	6.39 (1.31)

**Table 3:** Hierarchical binary logistic regression table for absolutist level.

Step	Predictors	B	S.E.	Wald	df	*p*	OR	95% CI for OR	
1	Family Income	−0.53	0.27	3.81	1	0.051	0.59	0.35	1.00
	Constant	0.27	0.15	3.46	1	0.063	1.31		
2	Family Income	−0.23	0.29	0.65	1	0.420	0.79	0.45	1.40
	Asian	−0.19	0.60	0.10	1	0.747	0.83	0.26	2.66
	Black or African American	0.86	0.37	5.41	1	0.020	2.37	1.15	4.90
	Hispanic or Latino	0.95	0.31	9.21	1	0.002	2.57	1.40	4.74
	Constant	−0.41	0.27	2.24	1	0.135	0.66		

$\chi^{2}$(4) = 16.14, *p* = 0.003, $R^{2}$ = 0.06 (Cox–Snell), 0.08 (Nagelkerke).

**Table 4:** Hierarchical binary logistic regression table for multiplist level.

Step	Predictors	B	S.E.	Wald	df	*p*	OR	95% CI for OR	
1	Family Income	−2.24	1.03	4.68	1	0.030	0.11	0.01	0.81
	Constant	−2.11	0.23	83.28	1	<0.001	0.12		
2	Family Income	−2.01	1.05	3.69	1	0.055	0.13	0.02	1.04
	Asian	−18.08	10,006.33	0.00	1	0.999	0.00	0.00	.
	Black or African American	0.74	0.75	0.99	1	0.321	2.10	0.49	9.02
	Hispanic or Latino	0.69	0.67	0.99	1	0.320	1.95	0.52	7.29
	Constant	−2.63	0.61	18.49	1	<0.001	0.07		

$\chi^{2}$(4) = 12.67, *p* = 0.013, $R^{2}$ = 0.05 (Cox–Snell), 0.11 (Nagelkerke).

**Table 5:** Hierarchical binary logistic regression table for evaluativist level.

Step	Predictors	B	S.E.	Wald	df	*p*	OR	95% CI for OR	
1	Family Income	0.94	0.27	11.68	1	<0.001	2.56	1.49	4.38
	Constant	−0.73	0.15	22.80	1	<0.001	0.48		
2	Family Income	0.60	0.30	4.14	1	0.042	1.83	1.02	3.26
	Asian	0.40	0.60	0.44	1	0.508	1.49	0.46	4.83
	Black or African American	−1.09	0.39	8.04	1	0.005	0.34	0.16	0.71
	Hispanic or Latino	−1.16	0.32	13.35	1	<0.001	0.31	0.17	0.58
	Constant	0.07	0.27	0.07	1	0.796	1.07		

$\chi^{2}$(4) = 31.13, *p* < 0.001, $R^{2}$ = 0.11 (Cox–Snell), 0.15 (Nagelkerke).

## Data Availability

The raw data supporting the conclusions of this article will be made available by the authors on request.

## Abbreviation

The following abbreviation is used in this manuscript:

ETA	Epistemic Thinking Assessment
